# Altered oral microbiota and immune dysfunction in Chinese elderly patients with schizophrenia: a cross-sectional study

**DOI:** 10.1038/s41398-023-02682-1

**Published:** 2023-12-09

**Authors:** Zongxin Ling, Yiwen Cheng, Xia Liu, Xiumei Yan, Lingbin Wu, Li Shao, Jie Gao, Wenhui Lei, Qinghai Song, Longyou Zhao, Guolin Jin

**Affiliations:** 1 Collaborative Innovation Center for Diagnosis and Treatment of Infectious Diseases, State Key Laboratory for Diagnosis and Treatment of Infectious Diseases, National Clinical Research Center for Infectious Diseases, the First Affiliated Hospital, School of Medicine, Zhejiang University, 310003 Hangzhou, Zhejiang China; 2 Jinan Microecological Biomedicine Shandong Laboratory, 250000 Jinan, Shandong China; 3 Department of Intensive Care Unit, the First Affiliated Hospital, School of Medicine, Zhejiang University, 310003 Hangzhou, Zhejiang China; 4 Department of Laboratory Medicine, Lishui Second People’s Hospital, 323000 Lishui, Zhejiang China; 5 School of Clinical Medicine, Institute of Hepatology and Metabolic Diseases, Hangzhou Normal University, The Affiliated Hospital of Hangzhou Normal University, 310015 Hangzhou, Zhejiang China; 6 School of Basic Medicine, Shandong First Medical University, 250000 Jinan, Shandong China; 7 Department of Psychiatry, Lishui Second People’s Hospital, 323000 Lishui, Zhejiang China

**Keywords:** Schizophrenia, Diagnostic markers

## Abstract

Schizophrenia (SZ) is a complex psychiatric neurodevelopmental disorder with uncertain etiology and pathogenesis. Increasing evidence has recognized the key role of the gut microbiota in SZ. However, few studies have investigated the potential link between oral microbiota and SZ. We studied the tongue coating microbiota and inflammatory profiles of 118 elderly SZ patients and 97 age-matched healthy controls using Illumina MiSeq sequencing and multiplex immunoassays, respectively. Reduced α-diversity, along with a significant difference in β-diversity, were observed in patients with SZ. We have identified SZ-associated oral dysbiosis, characterized by increased *Streptococcus* and *Fusobacterium*, as well as decreased *Prevotell*a and *Veillonella*. These differential genera could potentially serve as biomarkers for SZ, either alone or in combination. Additionally, an elevated *Streptococcus/Prevotella* ratio could indicate oral dysbiosis. These differential genera formed two distinct clusters: *Streptococcus*-dominated and *Prevotella*-dominated, which exhibited different correlations with the altered immunological profiles. Furthermore, we also observed disruptions in the inferred microbiota functions in SZ-associated microbiota, particularly in lipid and amino acid metabolism. Our study provides novel insights into the characteristics of tongue coating microbiota and its associations with immunological disturbances in elderly SZ patients, which offer new targets for the diagnosis and treatment of SZ in the elderly.

## Introduction

Schizophrenia (SZ) is a severe neuropsychiatric disorder characterized by the persistence of symptoms throughout adult life in most of the affected patients. The typical onset of SZ occurs mostly in late adolescence to early adulthood, with a peak in prevalence at approximately 40 years of age, which appears as an inverted U-shaped shape [1]. With the significant population growth and aging, the number of elderly patients with SZ has increased significantly. Owing to the epidemiological characteristics of SZ, it can be easily overlooked in the elderly and may be mistaken for other age-related neurodegenerative disorders. While current therapies for SZ can help alleviate delusions and hallucinations, they do not substantially improve the overall outcome of this highly incapacitating illness. Thus, there is an urgent need to understand the etiology and underlying mechanisms of SZ, as well as develop novel and effective therapeutic options for its treatment.

Mounting evidence suggests that both genetic and environmental factors are associated with the onset of SZ [2]. As an essential environmental factor, scientists have found that the gut microbiota may be involved in the development and progression of adult SZ through the gut-brain axis [3–16]. In our previous study, we observed structural and functional dysbiosis of the gut microbiota in elderly Chinese patients with SZ, which could potentially contribute to immune disturbances in the host [17]. Immune-related abnormalities, such as higher levels of pro-inflammatory cytokines in peripheral serum, have been found to play a significant role in the pathogenesis of SZ [18, 19]. Like the gut microbiota, the oral microbiota contributes to maintaining both local and systemic health, which has recently attracted considerable attention due to its potential role in psychiatric disorders and its ability to modulate host immunity. The oral microbiota serves as the entry point for microorganisms into the body and is a direct precursor of oral diseases. Dysbiosis in the oral microbiota can have systemic effects, leading to various diseases throughout the body [20]. Disruptions in the oral microbiota can contribute to gut dysbiosis and immune system disorders, which in turn can disrupt brain function and contribute to the development of neuropsychiatric diseases [21]. Accumulating evidence indicates the involvement of oral microbiota in the pathogenesis of adult SZ [22–24]. Recently, Lee et al. have demonstrated that the oral microbiome is significantly altered in adult SZ patients, and the differential bacteria can accurately classify psychiatric phenotypes. The correlations between microbiota and inflammatory markers in the periphery and hippocampus identify potential oral-brain axis mechanisms [25]. In addition, the human microbiota undergoes a progressive, age-related physiological succession of species across its life cycle, and the aging microbiota is distinct from other healthy age groups [26]. Although the associations between SZ and the oral microbiota in adults have been studied, comprehensive knowledge about their relationship in the elderly is still lacking. To address this research gap, we aimed to explore the characteristics of oral microbiota profiles using tongue-coated swabs from elderly SZ patients using the 16S rRNA gene high-throughput MiSeq platform. Furthermore, we investigated the correlations between key SZ-associated oral functional bacteria and inflammatory cytokines. These analyses would provide insight into the association between tongue coating microbiota and elderly SZ and could potentially aid in the development of new precautionary or non-invasive diagnostic tools for elderly Chinese patients with SZ.

## Materials and methods

### Participants of the study

We recruited 118 well-controlled Chinese elderly SZ patients (age > 62 years) from Lishui, Zhejiang province (China) from April 2021 to August 2021, and 97 age- and gender-matched cognitively normal healthy controls. SZ was diagnosed based on the criteria of the Diagnostic and Statistical Manual of Mental Disorders Fourth Edition. The psychiatric symptoms of the SZ patients remained steady for more than 2 weeks; the Positive and Negative Syndrome Scale (PANSS) evaluated the rate of change <20% in 2 weeks and the total score of PANSS >30. The sample collection and analysis protocol were approved by the Ethics Committee of Lishui Second People’s Hospital (reference no.: 20180705-1), and written informed consent was obtained from each participant or their guardian before enrollment. The inclusion and exclusion criteria were as previously described in our previous study [17]. Detailed demographic data and medical history were collected using a set of questionnaires (Supplementary Table S1). There were no significant differences in age, sex, BMI, education, smoking, or drinking between the healthy controls and the elderly SZ patients (*p* > 0.05).

### Sample collection and bacterial DNA extraction

Using sterile cotton swabs, we obtained three swabs from the middle site of the tongue 20 times from each participant, following previously developed methods [27]. The swabs were collected in sterile plastic cups. Additionally, serum samples were obtained from the participants using their fasting blood in the morning. These samples were prepared within 15 min and stored at −80 °C until use.

### DNA extraction, MiSeq sequencing and bioinformatic analysis

Bacterial genomic DNA was extracted from the oral swabs, and amplicon library construction and sequencing were conducted following the methods described in our previous studies [17, 28–30]. MiSeq sequencing and library construction were performed by technical staff at Hangzhou KaiTai Bio-lab. The raw sequencing data were imported into QIIME2 v2020.11 for microbiota diversity and abundance analysis. To analyze the differences in the composition of the oral microbiota at different taxonomic levels, we used the Statistical Analysis of Metagenomic Profiles (STAMP) software package v2.1.3 [31] and the linear discriminant analysis (LDA) effect size (LEfSe) method [32]. Those taxa with an average relative abundance of more than 0.01% were used for biomarker discovery (LDA score > 3.0; *p* < 0.05). Receiver-operating characteristic (ROC) analysis was performed using the pROC package v1.15.3 in R v3.6.0. BugBase was utilized to predict the phenotype of microorganisms based on normalized OTUs in the tongue coating microbiota [33]. Additionally, PiCRUSt v1.0.0 was employed to identify predicted gene families and associated pathways from inferred metagenomes of taxa of interest identified from the compositional analyses [33]. The sequence data from this study have been deposited in the GenBank Sequence Read Archive under the accession number PRJNA983408.

### Serum cytokines analyses

The systemic immune function of the participants was evaluated using a method similar to our previous study [17]. We quantified 27 cytokines and chemokines using a 27-plex magnetic bead-based immunoassay kit (Bio-Rad, CA, USA), following the manufacturer’s instructions. The results were expressed as picograms per milliliter (pg/mL) using standard curves integrated into the assay. The analysis was performed using Bio-Plex Manager v5.0 software, which provided reproducible intra- and inter-assay coefficient of variation (CV) values of 5–8% [7, 17, 28, 29].

### Statistical analysis

For continuous variables, we used either White’s nonparametric *t*-test, independent *t*-test, or Mann–Whitney *U*-test, depending on the data distribution and assumptions. Categorical variables were analyzed using either Pearson’s chi-square test or Fisher’s exact test to compare the proportions between groups. Correlation analyses were performed using Spearman’s rank correlation test. Statistical analysis was performed using the SPSS v24.0 (SPSS Inc., Chicago, IL) and STAMP v2.1.3 [31]. Graphs were prepared using R packages and GraphPad Prism v6.0. All tests of significance were two-sided, and p values were adjusted for multiple testing by using the Bonferroni method and a false discovery rate (FDR) <0.05 was considered statistically significant.

## Results

### Altered overall structure of the tongue coating microbiota in elderly patients with SZ

**Fig. 1:**
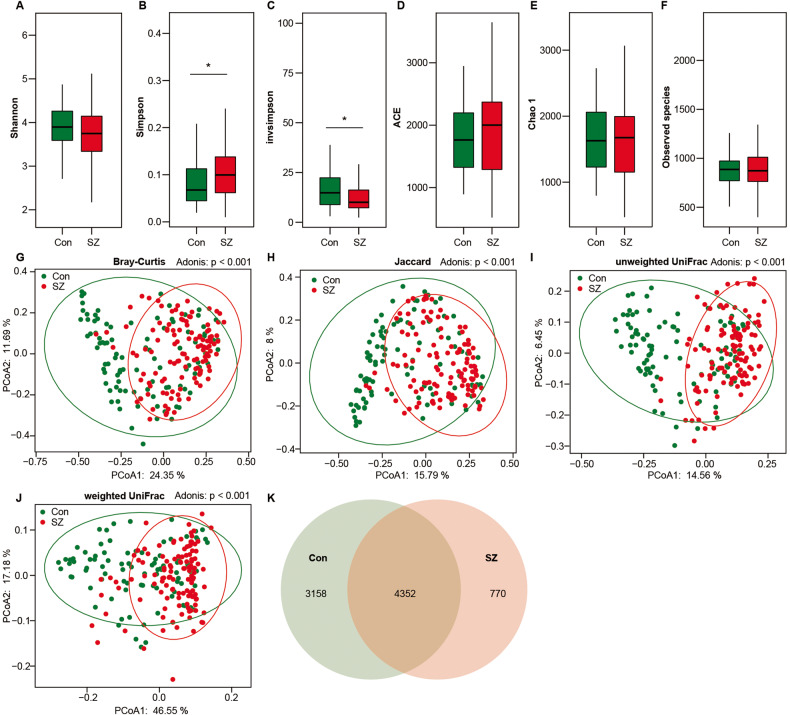
Altered overall structure of the tongue coating microbiota in elderly SZ patients. The diversity indices of Shannon (**A**), Simpson (**B**) and invsimpson (**C**), as well as the richness indices of ACE (**D**), Chao1 (**E**), and the observed species (**F**), were utilized to assess the overall structure of the tongue coating microbiota in SZ patients and healthy controls. The data are presented as mean ± standard deviation. Unpaired *t*-tests (two-tailed) were used to analyze the variation between the groups. Principal coordinate analysis (PCoA) plots illustrated individual tongue coating microbiota based on Bray–Curtis (**G**), Jaccard (**H**), and unweighted (**I**) and weighted (**J**) UniFrac distances in the elderly SZ patients and the healthy controls. Each symbol represented a sample. The Venn diagram illustrated the overlap of OTUs in the SZ-associated microbiota and healthy controls (**K**).

After merging and filtering, we generated 10,889,850 high-quality sequence reads, with an average of 50,650 reads per sample, for subsequent microbiota analyses. Specifically, we obtained 5,988,865 and 4,900,985 reads from the SZ and healthy control groups, respectively. Across the entire cohort, we identified 8280 bacterial operational taxonomic units (OTUs) with a Good’s coverage of 98.65%, indicating that the majority of bacteria were identified from the tongue coating microbiota. To investigate whether the overall microbiota composition differed between SZ patients and healthy controls (HCs), we conducted α-diversity and β-diversity analyses. Using the OTU relative table, we estimated microbial diversity in the samples using α-diversity indices such as Shannon, Simpson, and invsimpson (inverse of Simpson) (Fig. 1A–C). Simpson and invsimpson revealed lower diversity in elderly SZ patients, while richness indices such as ACE, Chao1, and observed species were not significantly different between the two groups (Fig. 1D–F). Bacterial β-diversity, i.e., PCoA analysis based on Bray–Curtis, Jaccard, unweighted UniFrac, and weighted UniFrac phylogenetic distances, found a significant difference in composition between the two groups. This could divide the elderly SZ patients and the controls into two different clusters despite significant interindividual variations (ADONIS test: *p* < 0.01; Fig. 1G–J). Additionally, the Venn diagram showed more unique bacterial phylotypes in the controls (3158 OTUs) than in patients with SZ (770 OTUs), which was consistent with the changing trend in bacterial α-diversity (Fig. 1K). Overall, our findings suggested an altered overall structure of the tongue coating microbiota in elderly patients with SZ.

### Changed composition of the tongue coating microbiota in the elderly patients with SZ

**Fig. 2:**
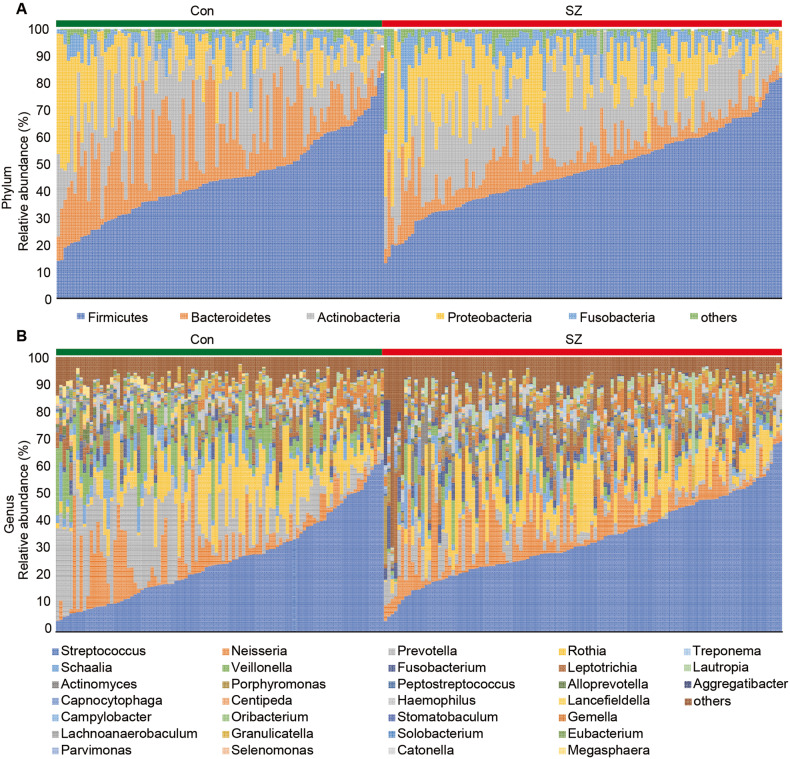
Variations in the tongue coating microbial composition in elderly SZ patients and healthy controls. Relative proportions of bacterial phylum (**A**) and genus (**B**) in elderly SZ patients (*n* = 118) and healthy controls (*n* = 97).

**Fig. 3:**
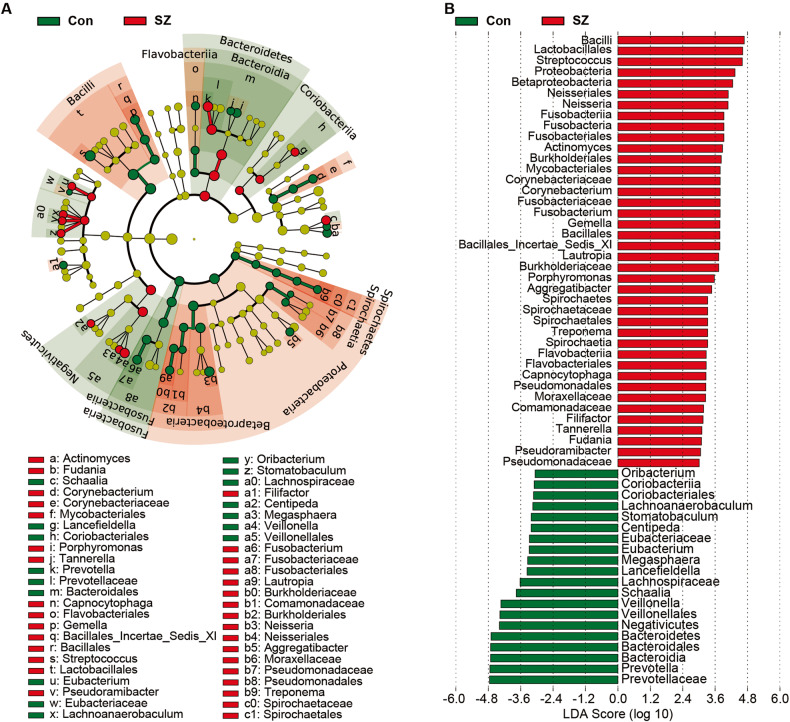
Differential bacterial taxa in the tongue coating microbiota between the elderly SZ patients and the healthy controls. **A** LEfSe cladograms showing the taxa most differentially associated with Control (green) or SZ (red) (Wilcoxon rank-sum test, *p* < 0.05). Circle sizes in the cladogram plot are proportional to bacterial abundance. The circles represent, going from the inner to outer circle: phylum, genus, class, order, family, and genus. **B** the histogram of LDA value distribution (LDA Score > 3.0) shows the taxa with the greatest differences in abundance between the SZ patients and controls (*p* < 0.05).

**Fig. 4:**
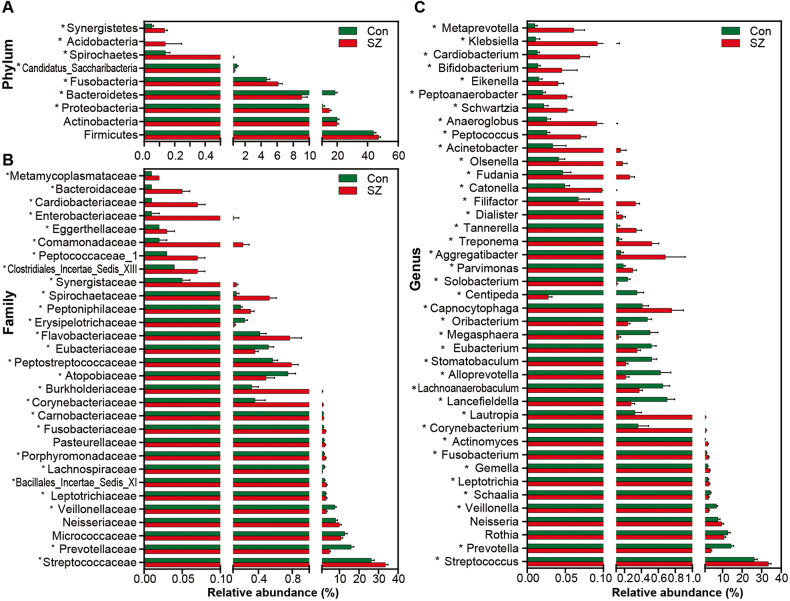
Metastats analysis of thetongue coating microbiota. Comparisons of the relative abundance of the abundant bacterial taxa at the level of bacterial phylum (**A**), family (**B**), and genus (**C**) in the tongue-coating microbiota. The data are presented as the mean ± standard deviation. Mann–Whitney *U*-tests were used to analyze variation between the elderly SZ patients and the healthy controls. *p < 0.05 compared with the control group.

**Fig. 5:**
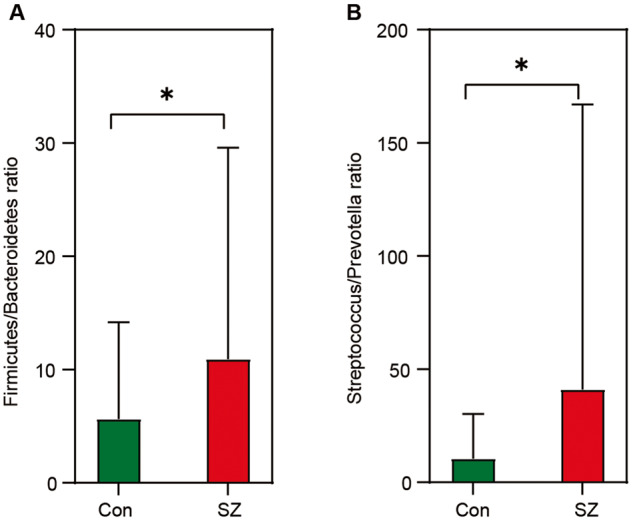
Potential dysbioticbiomarkers of the tongue-coating microbiota. Comparison of the ratio of Firmicutes/Bacteroidetes (**A**) and Streptococcus/Prevotella (**B**) in the tongue-coating microbiota between the elderly SZ patients and the healthy controls. White’s nonparametric *t* test was used; *p < 0.05.

Figure 2 illustrates the taxonomic composition and variation in the tongue coating microbiota at different taxonomic levels. Using LEfSe, several key functional differential taxa were identified between SZ patients and HCs (LDA score > 3, *p* < 0.05; Fig. 3). The five predominant phyla, including Firmicutes, Actinobacteria, Proteobacteria, Bacteroidetes, and Fusobacteria, accounted for more than 97% of the total sequences. SZ patients had higher levels of Proteobacteria, Fusobacteria, Spirochaetes, Acidobacteria, and Synergistetes, and lower levels of Bacteroidetes and Candidatus_Saccharibacteria than HCs (Fig. 4A), while the Firmicutes/Bacteroidetes ratio was significantly higher in SZ patients (*p* < 0.05; Fig. 5A). At the family level, four families, including Prevotellaceae, Veillonellaceae, Atopobiaceae, and Erysipelotrichaceae, were decreased, whereas the other 23 families, including Streptococcaceae and Leptotrichiaceae, were enriched in SZ patients (Fig. 4B). At the genus level, 39 genera were found to be differentially expressed between SZ patients and HCs (Fig. 4C). Among these differential oral genera, the proportions of 12 genera, including *Prevotella*, *Veillonella*, *Schaalia*, *Lancefieldella*, *Lachnoanaerobaculum*, *Alloprevotella*, *Stomatobaculum*, *Eubacterium*, *Megasphaera*, *Oribacterium*, *Centipeda*, and *Solobacterium*, were reduced in patients with SZ, whereas the other 27 genera, including *Streptococcus* and *Leptotrichia*, were significantly increased. The *Streptococcus/Prevotella* ratio was also significantly higher in SZ patients (*p* < 0.05; Fig. 5B), suggesting that this ratio could be used as a potential biomarker to discriminate SZ-associated oral dysbiosis. Based on our present findings, the dysbiosis of the tongue coating microbiota was evident in elderly patients with SZ.

**Fig. 6:**
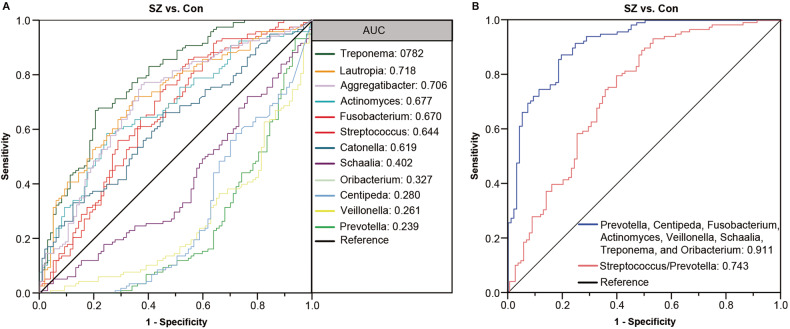
The differential genera as elderly SZ diagnostic markers. Receiver-operating characteristic (ROC) curves for the differential genera alone (**A**) or in combination (**B**) were used to discriminate elderly SZ patients from healthy controls. AUC is the area under the receiver-operating characteristic curve.

To explore the potential usefulness of these key functional differential genera as non-invasive diagnostic biomarkers for elderly patients with SZ, ROC analyses were performed. The area under the ROC curve (AUC) was used to evaluate the specificity and sensitivity of the characteristic tongue coating microbiota for diagnosing SZ in elderly individuals. Based on the LEfSe analysis, several genera showed potential as biomarkers to discriminate SZ patients from HCs. The discriminating value of several genera, including *Treponema*, *Lautropia*, *Aggregatibacter*, *Actinomyces*, *Fusobacterium*, *Streptococcus*, *Catonella*, *Schaalia*, *Oribacterium*, *Centipeda*, *Veillonella*, and *Prevotella*, was assessed. When using each of these key functionally differential bacteria as a predictor individually, ROC-AUC values ranging from 0.239 to 0.782 were obtained, demonstrating their predictive value (Fig. 6A). The data indicated that enriched *Treponema*, *Lautropia*, and *Actinomyces* in SZ patients were effective discriminant predictors for SZ (AUC > 0.700), while decreased *Centipeda*, *Veillonella*, and *Prevotella* in SZ patients were useful discriminant predictors for HCs (AUC > 0.700). To improve the predictive value for SZ, a multivariable stepwise logistic regression analysis was applied to the list of SZ-associated genera to determine the taxa that best distinguished SZ patients from HCs. A predictive model consisting of seven differential genera, namely *Prevotella*, *Centipeda*, *Fusobacterium*, *Actinomyces*, *Veillonella*, *Schaalia*, and *Treponema* (Fig. 6B). The AUC for this model was 0.911, indicating good predictive performance. Additionally, the *Streptococcus/Prevotella* ratio, an indicator of the oral microbiota, also distinguished SZ patients from HCs (AUC = 0.743). These findings suggest that these key functionally differential genera, either alone or in combination, could serve as biomarkers to discriminate SZ patients from HCs in our cohort.

### Changes in inferred microbial function

To predict microbial phenotypes at the organism level, we analyzed the tongue coating microbiota using BugBase. BugBase is a novel method that provides biologically relevant predictions of microbial phenotypes. We performed pairwise Mann–Whitney-Wilcoxon tests to compare the phenotypes between elderly SZ patients and controls. The results showed a significant decrease in anaerobic status in elderly SZ patients (*p* < 0.05). However, no significant differences were observed in gram-positive stats, gram-negative stats, aerobic stats, mobile element stats, facultative anaerobic stats, biofilm stats, potentially pathogenic states, and stress tolerance between SZ patients and HCs.

**Fig. 7:**
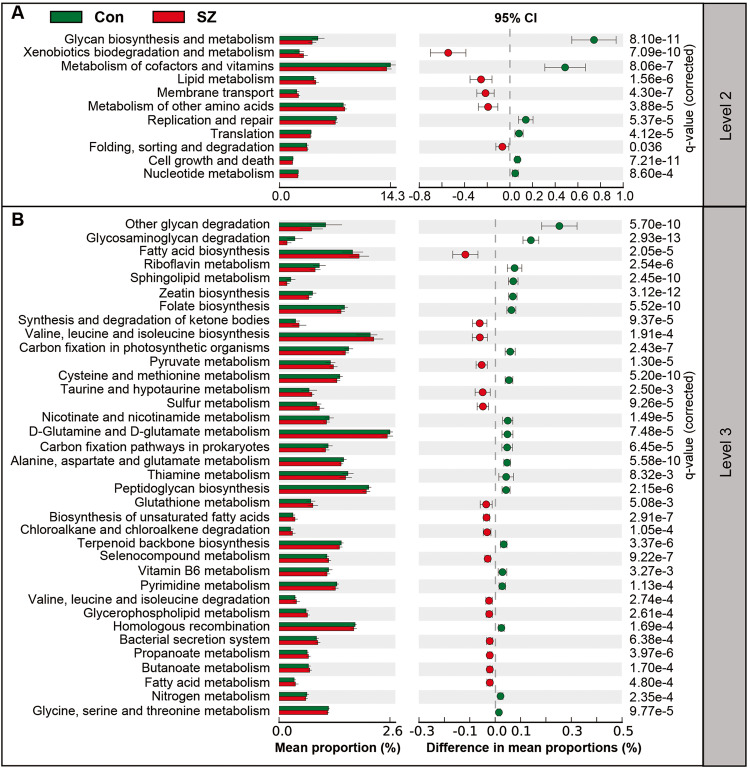
PiCRUSt-based examination of the tongue coating microbiota of the elderly SZ patients and the healthy controls. The different bacterial functions were evaluated between the two groups based on two-sided Welch’s *t*-test. The comparisons between the groups for each KEGG functional category at level 2 (**A**) and level 3 (**B**) are shown by percentage. The Benjamini–Hochberg method was used for multiple testing correction based on the false discovery rate (FDR) through STAMP.

Furthermore, we used PiCRUSt to predict gene functions in the tongue coating microbiota and compared the results between the two groups (Fig. 7). PiCRUSt analysis was performed based on closed-reference OTUs to determine changes in predicted microbial functions. We compared 64 Kyoto Encyclopedia of Genes and Genomes (KEGG) pathways at level 2 and identified 11 differential categories between SZ patients and HCs. Microbes with higher relative abundances in HCs were associated with functions related to cofactor and vitamin metabolism, glycan biosynthesis and metabolism, replication and repair, and cell growth and death. On the other hand, five KEGG pathways, including xenobiotics biodegradation and metabolism, lipid metabolism, membrane transport, metabolism of other amino acids, as well as folding, sorting, and degradation, were significantly increased in SZ patients (*q* < 0.05). Additionally, 36 discernible microbiota pathways at level 3 showed significant differences between the two groups (*q* < 0.05). Notably, 16 pathways, such as fatty acid biosynthesis, valine, leucine, and isoleucine biosynthesis, and biosynthesis of unsaturated fatty acids, exhibited higher activity in the SZ-associated microbiota. Conversely, 20 pathways, including glycosaminoglycan degradation, D-Glutamine and D-glutamate metabolism, and peptidoglycan biosynthesis, showed prominently decreased activity. Taken together, the functional changes observed in the tongue coating microbiota suggest that the oral microbiota may play a role in the pathogenesis and development of SZ in elderly individuals.

### Associations between key functional genera and host immunological profiles

**Fig. 8:**
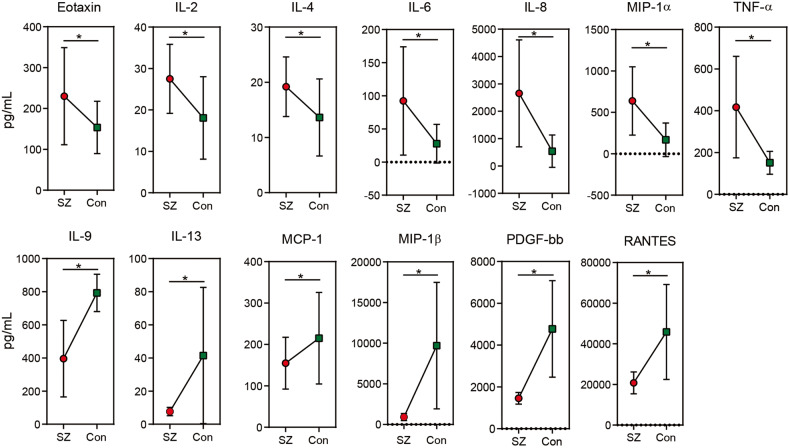
SZ-associated immune dysfunction in elderly SZ patients. The mean concentrations (pg/ml) of 27 pro- and anti-inflammatory cytokines, chemokines, and growth factors in the elderly SZ patients and in healthy controls were determined using Bio-Plex immunoassays. In the elderly SZ patients, there was a significant increase in the concentrations of Eotaxin, IL-2, IL-4, IL-6, IL-8, MIP-1α and TNF-α, while a significant decrease was observed in the concentrations of IL-9, IL-13, MCP-1, MIP-1β, PDGF-bb and RANTES decreased significantly. Unpaired *t*-test was used; *: *p* < 0.05.

**Fig. 9:**
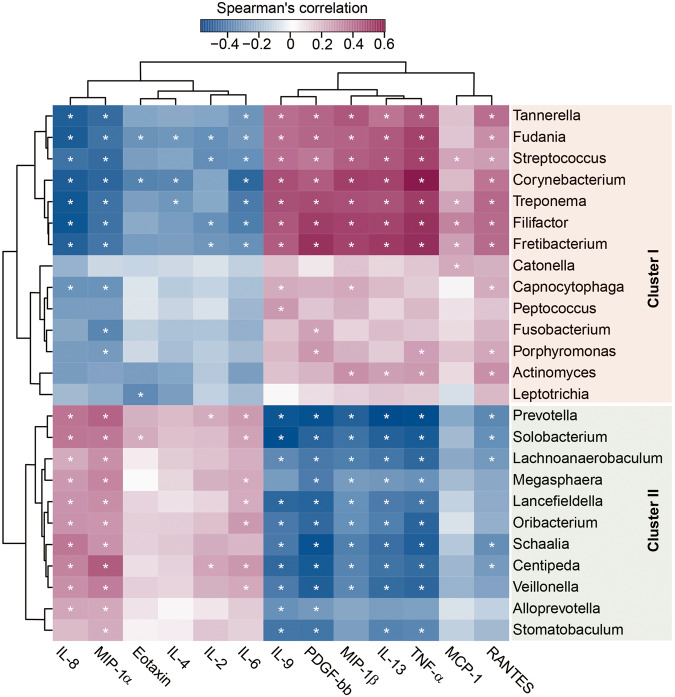
Correlation between key functional differential genera and pro- and anti-inflammatory cytokines and chemokines. The heatmap shows partial Spearman’s correlation coefficients between key functional genera and host immunity in SZ patients, categorized into two distinct clusters: *Streptococcus*-dominated and *Prevotella*-dominated. Spearman’s rank correlation (r) and probability (*p*) were used to evaluate statistical importance. *: *p* < 0.01.

The serum immunological profiles were analyzed using a Bio-Plex Pro Human Cytokine 27-Plex Assay on a Bio-Rad MAGPIX Multiplex Reader to investigate associations between key functional genera and host immunological profiles. The cytokine profiles of SZ patients and HCs were compared, revealing patterns consistent with our previous study. Specifically, the levels of Eotaxin, IL-2, IL-4, IL-6, IL-8, MIP-1α, and TNF-α were significantly increased in SZ patients, while the concentrations of IL-9, IL-13, MCP-1, MIP-1β, and RANTES were significantly decreased (Fig. 8, *p* < 0.05 for each). As changes in the oral microbiota can contribute to systemic inflammation, we conducted correlation analyses between serum cytokines and key functional differential genera. The correlations between the cytokines and key functional genera were divided into four groups (Fig. 9). *Streptococcus* was the dominant genus in cluster I, while *Prevotella* was the dominant genus in cluster II. These enriched differential genera in elderly SZ patients were positively associated with the increased cytokines, mainly pro-inflammatory cytokines and chemokines such as TNF-α, and negatively correlated with the decreased cytokines, mainly anti-inflammatory cytokines in the *Streptococcus*-dominated cluster. Conversely, the reduced genera in the *Prevotella*-dominated cluster showed opposite correlations with these immunological profiles, suggesting that the SZ-associated microbiota cluster I exhibited pro-inflammatory effects, while cluster II demonstrated anti-inflammatory effects. Overall, our findings suggest that different clusters of oral microbiota in elderly SZ patients may have distinct roles in modulating the immune response and contributing to the pathophysiology of elderly SZ.

## Discussion

Accumulating evidence has increasingly recognized the gut microbiota as a crucial link between the gut and brain. Disturbances in the microbiota-gut-brain axis have been implicated in the pathophysiology of several psychiatric disorders, including SZ, with microbiota signatures being proposed as potential biomarkers [34–36]. Over the past decade, significant progress has been made in understanding the roles and mechanisms of the gut microbiota in psychiatric disorders. Similarly, the oral microbiota has emerged as a new area of research focused on exploring microbiota-brain interactions. The oral cavity harbors the second most abundant microbial symbionts after the gastrointestinal tract [37]. It contains approximately 50–100 billion bacteria and 600 prevalent taxa at the species level, which play a crucial protective role against the colonization of external bacteria that can impact overall health [38]. As the oral cavity serves as the first organ of the gastrointestinal tract, the oral microbiota influences the diversity and composition of the gut microbiota and affects brain function, thereby extending the concept of the microbiota-gut-brain axis to the microbiota-oral-brain axis. Recent studies have also reported connections between the oral microbiota and various brain disorders [21–23, 39–42]. Oral bacteria can migrate from the mouth to the brain and influence neuroimmune activity and inflammation through several plausible pathways [40, 43]. Dysbiosis of the oral microbiota has emerged as an etiological factor in brain disorders. Similar to the gut microbiota, the oral microbiota may play a pivotal role in the complex chain of SZ development.

SZ is a severe and chronic psychiatric disorder that causes significant personal and societal burdens due to its severe and long-term disability. Promoting disease diagnosis, complementing disease treatment, developing personalized medicines, and improving disease prognosis have become major challenges for psychiatrists and scientists. Previous studies have investigated the role of the oral microbiota in the pathogenesis of SZ by targeting the microbiota-oral-brain axis. However, current studies have not reached a consensus on the changing patterns of the SZ-associated oral microbiota. Reports attribute this disparity to differences in geography, genotype, diet, and lifestyle among patients with SZ. This study is the first to describe the altered oral microbiota and immunological profiles among elderly Chinese SZ patients in a larger cohort, providing new insights into the pathogenesis of SZ. The tongue coating is an important habitat of the oral cavity, and it is relatively easy and non-invasive to collect for microbiota analysis. Additionally, tongue coating has high sampling stability, which makes it a more stable and representative sample of the oral microbiota than saliva or plaque [44]. The tongue coating microbiota exhibits stability with a moderate rate of shedding of biofilms formed by tongue epithelial cells and microbiota, making it an ideal site for study [45]. The composition of the abundant microbiota in tongue coating can reflect some personalized features and disease conditions of the body [46].

Our study found significant changes in the overall structure and composition of the tongue coating microbiota in elderly SZ patients. Along with significant alterations in bacterial β-diversity, several key functional differential taxa were identified. Predicting the microbial phenotypes at the organism level by BugBase revealed a significant decrease in the proportion of anaerobic bacteria in elderly patients with SZ, indicating that the altered oral microenvironment may affect the anaerobic/aerobic proportion. Compared to age-matched elderly HCs, the most striking differences in SZ were higher proportions of Proteobacteria and Fusobacteria and a reduction in Bacteroidetes. The Firmicutes/Bacteroidetes ratio, a biomarker of gut dysbiosis, was significantly increased in the oral microbiota of SZ patients, suggesting oral dysbiosis in elderly patients with SZ [17]. Several predominant genera, including *Streptococcus* (belonging to Firmicutes) and *Prevotella* (belonging to Bacteroidetes), showed significant changes in the elderly SZ patients. Interestingly, the changing patterns observed in the oral cavity were inconsistent with our findings in the fecal microbiota of the same group of elderly SZ patients. In our previous study, we observed an increased relative abundance of *Streptococcus* and *Prevotella* in the fecal microbiota of elderly SZ patients [17]. However, in the tongue coating microbiota, we found a higher proportion of *Streptococcus* and a reduction in *Prevotella*. Notably, the two most predominant genera in the oral microbiota, *Streptococcus* and *Prevotella*, exhibited a co-exclusion interaction, whereas both genera demonstrated a co-existence interaction in the fecal microbiota. Previous studies have recognized the *Streptococcus/Prevotella* ratio as an important defining characteristic across different esophageal community types [47, 48]. Additionally, we discovered that an increased *Streptococcus/Prevotella* ratio could serve as a useful indicator of SZ-associated oral dysbiosis for the first time. These findings suggest that a higher *Streptococcus/Prevotella* ratio is a risk factor for the development of SZ in elderly individuals.

*Streptococcus*, a potentially pathogenic genus found in the tongue coating microbiota, accounted for approximately 33% of the oral microbiota associated with SZ, compared to only 26% in the healthy controls. We also observed an increased relative abundance of *Streptococcus* in both the oral and fecal microbiota, suggesting that the enriched *Streptococcus* may translocate from the oral cavity to the gastrointestinal tract. This finding aligns with a previous study that reported a higher prevalence of oral bacterial transition to the gut in the elderly population compared to adults [49]. A case-control study utilizing metagenomic shotgun sequencing revealed that SZ patients had a higher abundance of bacteria typically found in the oral cavity, such as *S. salivarius*, compared to the healthy controls. This finding suggests a connection between the oral and gut microbiota in SZ patients [4, 50]. The enrichment of *Streptococcus* in both the oral cavity and gastrointestinal tract implies its potential pathogenic role in elderly SZ patients. *Streptococcus* is one of the most prevalent genera within the SZ-associated pathogenic microbiota cluster I, and it contributes to elevated levels of inflammatory markers like TNF-α and IL-9 in elderly SZ patients. This indicates that oral *Streptococcus* acts as a potent immunogenic trigger. In contrast to the relationship between gut microbiota and inflammation, the translocation of oral microbiota into the bloodstream may lead to chronic low-grade inflammation and increased levels of pro-inflammatory cytokines in SZ. The pro-inflammatory cytokines induced by oral *Streptococcus* could compromise the integrity of the blood-brain barrier, allowing bacteria to reach the brain and contribute to the development of neurological diseases [51]. Previous studies have demonstrated that an overgrowth of *Streptococcus* in dental samples can lead to neurological damage through the production of neurotoxins, including streptomycin, streptodornase, and streptokinase. This overgrowth and toxin production increase the risk of brain dysfunction, such as Tourette Syndrome (another neurodevelopmental disorder), Sydenham chorea, and bacterial meningitis [52–54]. Within the oral *Streptococcus* spp., *S. mutans* is the primary pathogenic member of the oral microbiota and plays a role in the progression of periodontal diseases and dental caries [55]. The cariogenic bacterium *S. mutans* exhibited a significant positive correlation with the Hamilton scale [21], indicating a potential link between its abundance and the severity of symptoms in SZ patients. Additionally, other *Streptococcus* spp. isolated from the oral cavity, such as *S. parasanguinis* and *S. salivarius*, demonstrated strong positive associations with peripheral tryptophan levels [56]. The metabolism of tryptophan/kynurenine played a vital role in mediating glutamatergic neurotransmission, specifically through the N-methyl-D-aspartic acid (NMDA) receptor, and was associated with a pro-inflammatory immune response involving the activation of the enzyme indoleamine 2,3-dioxygenase (IDO). This process leads to an increased production of kynurenic acid in the brain and an imbalance in glutamatergic neurotransmission and ultimately resulting in NMDA antagonism observed in SZ [57]. Valles-Colomer et al. reported that *S. vestibularis* demonstrated a functional neuroactive potential in mice, which was associated with changes in animal behaviors [58]. Recently, Zhu et al. also demonstrated that transplantation of another species of *Streptococcus*, *S. vestibularis*, induced deficits in social behaviors and altered neurotransmitter levels in the peripheral tissues of recipient mice, emphasizing its importance in the development of SZ [4]. Other genera in Cluster I, including *Actinomyces*, *Porphyromonas*, *Leptotrichia*, *Fusobacterium*, and *Tannerella*, may play active roles in the development and progression of SZ, although their exact mechanisms and roles remain elusive. However, our ROC analysis of these genera alone or in combination effectively discriminated between patients with SZ and healthy controls. Based on our current findings, these oral functional genera in Cluster I could be considered putative pathobionts for elderly SZ and used as biomarkers for SZ diagnosis and targets for personalized SZ treatment.

Among the key functionally differential genera associated with SZ in Cluster II, *Prevotella* was identified as one of the representative genera in the oral cavity of elderly patients with SZ. Oral *Prevotella* constitutes a significant portion of oral microbial communities and appears to play important roles in the gastrointestinal and respiratory tracts during both health and disease [59, 60]. In contrast to the oral *Streptococcus* genus, a reduction in *Prevotella* was observed in the tongue coating microbiota of individuals with SZ compared to controls. Previous studies have also noted the decreased abundance of oral *Prevotella* in several psychiatric disorders, including depression, autism spectrum disorders, schizophrenia, and mania [24, 61, 62]. Conversely, higher levels of *Prevotella* have been found in patients with Parkinson’s disease in Western countries [63], indicating that changes in oral *Prevotella* were influenced by lifestyle factors and diseases. In a case-control study, *Prevotella* was identified as one of the key members of a healthy oropharyngeal microbiota when compared to SZ patients [23]. Therefore, *Prevotella* could be considered a relatively non-pathogenic microorganism that might serve as a biomarker for a healthy oral cavity in the elderly. It was worth noting that different species of oral *Prevotella* may have distinct roles in the occurrence of mental illness, although there was limited availability of comparative analyses focusing on oral *Prevotella* species [64]. While there has been increasing interest in exploring the potential link between oral *Prevotella* and SZ, the specific roles and mechanisms of *Prevotella* in relation to SZ remain unclear. As the representative oral key functional differential genus in Cluster II, *Prevotella* could break down complex carbohydrates into short-chain fatty acids, with folate and thiamine as byproducts. These compounds may modulate the host immune response and inhibit peripheral inflammation in elderly SZ patients. Therefore, oral *Prevotella* appears to play a beneficial role in preventing the occurrence and development of SZ. However, in contrast to our present findings in the oral microbiota, a higher proportion of *Prevotella* in the gastrointestinal tract has been positively correlated with pro-inflammatory cytokines [17, 65]. Like other oral bacteria, oral *Prevotella* could colonize the lower gastrointestinal tract via a direct oral-fecal route and directly influence the community structure and function of the gut microbiota. However, compared to other oral bacteria, the impact of oral *Prevotella* spp. on the gut microbiota was still understudied. *Prevotella* was generally abundant in the gut of healthy Asian individuals, and it contributed to the digestion of a high-fiber diet [66], which could lead to the formation of a *Prevotella*-enterotype. Recent research has shown that SZ patients with a *Prevotella*-enterotype had an increased risk of obesity, indicating a significant influence on various metabolic pathways that disrupted the metabolism of glucose and lipids in the human body [67]. In contrast to our findings, Lee et al. found that an enrichment of *Prevotella* in the oral microbiota accurately classified psychiatric phenotypes [25]. Another study found a positive correlation between oral *Prevotella* species, such as *P. nigrescens*, and peripheral blood alanine levels, suggesting a direct communication pathway between the oral microbiota and the brain [21]. Furthermore, elderly SZ patients showed a significant decrease in the abundance of oral *Veillonella* spp. Oral *Veillonella*, along with other key functionally differential genera like *Schaalia* and *Oribacterium*, formed a second cluster of correlations with inflammatory cytokines. Due to their impact on the gut microbiota, neurotransmitters, and peripheral inflammation, oral *Prevotella* and the genera in Cluster II may play crucial roles in the development of SZ.

Our study presented a comprehensive survey and analysis of the SZ-associated microbiota and yielded several new findings. However, it is important to acknowledge several limitations in this study. First, although we enrolled a large number of participants as the discovery cohort, we did not include new subjects as a validation cohort. Future multicenter oral microbiota studies should incorporate validation cohorts to confirm and validate our findings. Second, our current sequencing technique only enables the identification of the oral microbiota at the genus level rather than the species level. Additionally, we were only able to correlate circulating cytokines with differential genera, which may result in the omission of crucial information when interpreting the oral microbiota associated with SZ. Conducting further metagenomic studies would provide more comprehensive and valuable insights into alterations in the oral microbiota among elderly SZ patients. Third, this study solely demonstrated an association between the oral microbiota and elderly SZ patients. Future multi-omics investigations of the oral microbiota in elderly SZ patients were encouraged to establish the causality between the oral microbiota and elderly SZ. Fourth, we did not specifically examine the relationship between SZ severity and the oral microbiota since all the SZ patients included in our study had well-controlled symptoms. Exploring such associations could provide additional insights into the complex dynamic interactions between SZ and the oral microbiota, contributing to our understanding of SZ pathophysiology and potential treatment strategies.

In summary, this study investigated the oral microbiota of a larger cohort of Chinese elderly SZ patients and identified key oral functional differential genera, such as *Streptococcus* and *Prevotella*, that formed two clusters correlated with peripheral inflammatory cytokines. These findings provide new insights into the potential role of oral dysbiosis in SZ pathogenesis and development. The differential genera could serve as putative biomarkers to distinguish between elderly SZ patients and controls, potentially improving the accuracy of SZ diagnosis in the elderly. Further research is needed to confirm these findings and explore the potential therapeutic implications of targeting the oral microbiota in SZ treatment or prevention.

### Supplementary information

Table S1

## Data Availability

The sequence data from this study have been deposited in the GenBank Sequence Read Archive (https://www.ncbi.nlm.nih.gov/sra) under the accession number PRJNA983408.

## References

[1] Charlson FJ, Ferrari AJ, Santomauro DF, Diminic S, Stockings E, Scott JG (2018). Global epidemiology and burden of schizophrenia: findings from the Global Burden of Disease Study 2016. Schizophr Bull.

[2] Ma L, Semick SA, Chen Q, Li C, Tao R, Price AJ (2020). Schizophrenia risk variants influence multiple classes of transcripts of sorting nexin 19 (SNX19). Mol Psychiatry.

[3] Zhu F, Guo R, Wang W, Ju Y, Wang Q, Ma Q (2020). Transplantation of microbiota from drug-free patients with schizophrenia causes schizophrenia-like abnormal behaviors and dysregulated kynurenine metabolism in mice. Mol Psychiatry.

[4] Zhu F, Ju Y, Wang W, Wang Q, Guo R, Ma Q (2020). Metagenome-wide association of gut microbiome features for schizophrenia. Nat Commun.

[5] Nguyen TT, Kosciolek T, Maldonado Y, Daly RE, Martin AS, McDonald D (2019). Differences in gut microbiome composition between persons with chronic schizophrenia and healthy comparison subjects. Schizophr Res.

[6] Li S, Song J, Ke P, Kong L, Lei B, Zhou J (2021). The gut microbiome is associated with brain structure and function in schizophrenia. Sci Rep.

[7] Ling Z, Zhu M, Liu X, Shao L, Cheng Y, Yan X (2020). Fecal fungal dysbiosis in Chinese patients with Alzheimer’s disease. Front Cell Dev Biol.

[8] Pan R, Zhang X, Gao J, Yi W, Wei Q, Su H (2020). Analysis of the diversity of intestinal microbiome and its potential value as a biomarker in patients with schizophrenia: a cohort study. Psychiatry Res.

[9] Pełka-Wysiecka J, Kaczmarczyk M, Bąba-Kubiś A, Liśkiewicz P, Wroński M, Skonieczna-Żydecka K (2019). Analysis of gut microbiota and their metabolic potential in patients with schizophrenia treated with olanzapine: results from a six-week observational prospective cohort study. J Clin Med.

[10] Ma X, Asif H, Dai L, He Y, Zheng W, Wang D (2020). Alteration of the gut microbiome in first-episode drug-naïve and chronic medicated schizophrenia correlate with regional brain volumes. J Psychiatr Res.

[11] Yuan X, Wang Y, Li X, Jiang J, Kang Y, Pang L (2021). Gut microbial biomarkers for the treatment response in first-episode, drug-naïve schizophrenia: a 24-week follow-up study. Transl Psychiatry.

[12] Fan Y, Gao Y, Ma Q, Yang Z, Zhao B, He X (2022). Multi-omics analysis reveals aberrant gut-metabolome-immune network in schizophrenia. Front Immunol.

[13] Munawar N, Ahmad A, Anwar MA, Muhammad K (2022). Modulation of gut microbial diversity through non-pharmaceutical approaches to treat schizophrenia. Int J Mol Sci.

[14] Xu R, Wu B, Liang J, He F, Gu W, Li K (2020). Altered gut microbiota and mucosal immunity in patients with schizophrenia. Brain Behav Immun.

[15] Ling ZX, Xiao H, Chen W (2022). Gut microbiome: the cornerstone of life and health. Adv Gut Microbiome Res..

[16] Murray N, Al Khalaf S, Bastiaanssen TFS, Kaulmann D, Lonergan E, Cryan JF (2023). Compositional and functional alterations in intestinal microbiota in patients with psychosis or schizophrenia: a systematic review and meta-analysis. Schizophr Bull.

[17] Ling Z, Jin G, Yan X, Cheng Y, Shao L, Song Q (2022). Fecal dysbiosis and immune dysfunction in Chinese elderly patients with schizophrenia: an observational study. Front Cell Infect Microbiol.

[18] Horváth S, Mirnics K (2014). Immune system disturbances in schizophrenia. Biol Psychiatry.

[19] Goldsmith DR, Rapaport MH, Miller BJ (2016). A meta-analysis of blood cytokine network alterations in psychiatric patients: comparisons between schizophrenia, bipolar disorder and depression. Mol Psychiatry.

[20] Tuganbaev T, Yoshida K, Honda K (2022). The effects of oral microbiota on health. Science.

[21] Krzyściak W, Karcz P, Bystrowska B, Szwajca M, Bryll A, Śmierciak N (2023). The association of the oral microbiota with the effects of acid stress induced by an increase of brain lactate in schizophrenia patients. Biomedicines.

[22] Qing Y, Xu L, Cui G, Sun L, Hu X, Yang X (2021). Salivary microbiome profiling reveals a dysbiotic schizophrenia-associated microbiota. NPJ Schizophr.

[23] Castro-Nallar E, Bendall ML, Pérez-Losada M, Sabuncyan S, Severance EG, Dickerson FB (2015). Composition, taxonomy and functional diversity of the oropharynx microbiome in individuals with schizophrenia and controls. PeerJ.

[24] Yolken R, Prandovszky E, Severance EG, Hatfield G, Dickerson F (2021). The oropharyngeal microbiome is altered in individuals with schizophrenia and mania. Schizophr Res.

[25] Lee JJ, Piras E, Tamburini S, Bu K, Wallach DS, Remsen B (2023). Gut and oral microbiome modulate molecular and clinical markers of schizophrenia-related symptoms: a transdiagnostic, multilevel pilot study. Psychiatry Res.

[26] Ling Z, Liu X, Cheng Y, Yan X, Wu S (2022). Gut microbiota and aging. Crit Rev Food Sci Nutr.

[27] Ren Z, Wang H, Cui G, Lu H, Wang L, Luo H (2021). Alterations in the human oral and gut microbiomes and lipidomics in COVID-19. Gut.

[28] Ling Z, Cheng Y, Yan X, Shao L, Liu X, Zhou D (2020). Alterations of the fecal microbiota in Chinese patients with multiple sclerosis. Front Immunol.

[29] Ling Z, Zhu M, Yan X, Cheng Y, Shao L, Liu X (2020). Structural and functional dysbiosis of fecal microbiota in Chinese patients with Alzheimer’s disease. Front Cell Dev Biol.

[30] Liu X, Shao L, Liu X, Ji F, Mei Y, Cheng Y (2019). Alterations of gastric mucosal microbiota across different stomach microhabitats in a cohort of 276 patients with gastric cancer. EBioMedicine.

[31] Parks DH, Tyson GW, Hugenholtz P, Beiko RG (2014). STAMP: statistical analysis of taxonomic and functional profiles. Bioinformatics.

[32] Segata N, Izard J, Waldron L, Gevers D, Miropolsky L, Garrett WS (2011). Metagenomic biomarker discovery and explanation. Genome Biol.

[33] Langille MG, Zaneveld J, Caporaso JG, McDonald D, Knights D, Reyes JA (2013). Predictive functional profiling of microbial communities using 16S rRNA marker gene sequences. Nat Biotechnol.

[34] Xie H, Zhang J, Gu Q, Yu Q, Xia L, Yao S (2023). Cohort profile: a prospective study of gut microbiota in patients with acute ischemic stroke. Adv Gut Microbiome Res..

[35] Wang J, Zhang P, Chen S, Duan H, Xie L (2022). Microbiota and gut health: promising prospects for clinical trials from bench to bedside. Adv Gut Microbiome Res..

[36] Sun J, Xu J, Ling Y, Wang F, Gong T, Yang C (2019). Fecal microbiota transplantation alleviated Alzheimer’s disease-like pathogenesis in APP/PS1 transgenic mice. Transl Psychiatry.

[37] Gunduz M, Murakami D, Gunduz I, Tamagawa S, Hiraoka M, Sugita G (2018). Recurrent bacterial translocation from gut and sepsis in Head and neck cancer patients and its prevention by probiotics. Med Hypotheses.

[38] Krishnan K, Chen T, Paster BJ (2017). A practical guide to the oral microbiome and its relation to health and disease. Oral Dis.

[39] Olsen I, Singhrao SK (2015). Can oral infection be a risk factor for Alzheimer’s disease?. J Oral Microbiol.

[40] Olsen I, Hicks SD (2020). Oral microbiota and autism spectrum disorder (ASD). J Oral Microbiol.

[41] Levert-Levitt E, Shapira G, Sragovich S, Shomron N, Lam JCK, Li VOK (2022). Oral microbiota signatures in post-traumatic stress disorder (PTSD) veterans. Mol Psychiatry.

[42] Niazi MK, Hassan F, Tufail T, ismail MA, Riaz K (2023). The role of microbiome in psychiatric diseases (insomnia and anxiety/depression) with microbiological mechanisms. Adv Gut Microbiome Res..

[43] Coureuil M, Lécuyer H, Bourdoulous S, Nassif X (2017). A journey into the brain: insight into how bacterial pathogens cross blood-brain barriers. Nat Rev Microbiol.

[44] Hall MW, Singh N, Ng KF, Lam DK, Goldberg MB, Tenenbaum HC (2017). Inter-personal diversity and temporal dynamics of dental, tongue, and salivary microbiota in the healthy oral cavity. NPJ Biofilms Microbiomes.

[45] Li Y, Cui J, Liu Y, Chen K, Huang L, Liu Y (2021). Oral, tongue-coating microbiota, and metabolic disorders: a novel area of interactive research. Front Cardiovasc Med.

[46] Wu J, Xu S, Xiang C, Cao Q, Li Q, Huang J (2018). Tongue coating microbiota community and risk effect on gastric cancer. J Cancer.

[47] Gall A, Fero J, McCoy C, Claywell BC, Sanchez CA, Blount PL (2015). Bacterial composition of the human upper gastrointestinal tract microbiome is dynamic and associated with genomic instability in a Barrett’s esophagus cohort. PLoS ONE.

[48] Deshpande NP, Riordan SM, Castaño-Rodríguez N, Wilkins MR, Kaakoush NO (2018). Signatures within the esophageal microbiome are associated with host genetics, age, and disease. Microbiome.

[49] Iwauchi M, Horigome A, Ishikawa K, Mikuni A, Nakano M, Xiao JZ (2019). Relationship between oral and gut microbiota in elderly people. Immun Inflamm Dis.

[50] Olde Loohuis LM, Mangul S, Ori APS, Jospin G, Koslicki D, Yang HT (2018). Transcriptome analysis in whole blood reveals increased microbial diversity in schizophrenia. Transl Psychiatry.

[51] Shoemark DK, Allen SJ (2015). The microbiome and disease: reviewing the links between the oral microbiome, aging, and Alzheimer’s disease. J Alzheimers Dis.

[52] Hornig M, Lipkin WI (2013). Immune-mediated animal models of Tourette syndrome. Neurosci Biobehav Rev.

[53] Libbey JE, Fujinami RS (2010). Role for antibodies in altering behavior and movement. Autism Res.

[54] Li W, Wu X, Hu X, Wang T, Liang S, Duan Y (2017). Structural changes of gut microbiota in Parkinson’s disease and its correlation with clinical features. Sci China Life Sci.

[55] Guo Y, Li B, Ma T, Moore ER, Xie H, Wu C (2022). Unraveling the binding microprocess of individual *Streptococcus mutans* cells via sucrose-dependent adhesion based on surface plasmon resonance imaging. J Oral Microbiol.

[56] Alkhalaf LM, Ryan KS (2015). Biosynthetic manipulation of tryptophan in bacteria: pathways and mechanisms. Chem Biol.

[57] Müller N (2018). Inflammation in schizophrenia: pathogenetic aspects and therapeutic considerations. Schizophr Bull.

[58] Valles-Colomer M, Falony G, Darzi Y, Tigchelaar EF, Wang J, Tito RY (2019). The neuroactive potential of the human gut microbiota in quality of life and depression. Nat Microbiol.

[59] Könönen E, Gursoy UK (2021). Oral *Prevotella* species and their connection to events of clinical relevance in gastrointestinal and respiratory tracts. Front Microbiol.

[60] Tett A, Pasolli E, Masetti G, Ercolini D, Segata N (2021). *Prevotella* diversity, niches and interactions with the human host. Nat Rev Microbiol.

[61] Wingfield B, Lapsley C, McDowell A, Miliotis G, McLafferty M, O’Neill SM (2021). Variations in the oral microbiome are associated with depression in young adults. Sci Rep.

[62] Qiao Y, Wu M, Feng Y, Zhou Z, Chen L, Chen F (2018). Alterations of oral microbiota distinguish children with autism spectrum disorders from healthy controls. Sci Rep.

[63] Zapała B, Stefura T, Milewicz T, Wątor J, Piwowar M, Wójcik-Pędziwiatr M (2022). The role of the western diet and oral microbiota in Parkinson’s disease. Nutrients.

[64] Ibrahim M, Subramanian A, Anishetty S (2017). Comparative pan genome analysis of oral *Prevotella* species implicated in periodontitis. Funct Integr Genomics.

[65] Yan F, Xia L, Xu L, Deng L, Jin G (2022). A comparative study to determine the association of gut microbiome with schizophrenia in Zhejiang, China. BMC Psychiatry.

[66] Kovatcheva-Datchary P, Nilsson A, Akrami R, Lee YS, De Vadder F, Arora T (2015). Dietary fiber-induced improvement in glucose metabolism is associated with increased abundance of *Prevotella*. Cell Metab.

[67] Liang Y, Shen Y, Li G, Yuan Y, Zhang M, Gao J (2022). Schizophrenia patients with *Prevotella*-enterotype have a higher risk of obesity. Front Psychiatry.

