# Supplementary material for: Altered oral microbiota and immune dysfunction in Chinese elderly patients with schizophrenia: a cross-sectional study
Source: Transl Psychiatry. 2023 Dec 9;13:383. doi: 10.1038/s41398-023-02682-1 (PMC10710460; doi:10.1038/s41398-023-02682-1)
Supplement: Supplementary file 1 — Table S1 [file 41398_2023_2682_MOESM1_ESM.docx]

**Table S1 Demographic characteristics of the participants of schizophrenia and health controls.**

|  | **SZ patients (n=118)** | **Control (n=97)** |
| --- | --- | --- |
| Age (y) | 75.18±10.14 | 74.68±9.91 |
| Gender (male/female) | 64/54 | 49/48 |
| BMI (Mean ± SD) | 23.60±2.58 | 24.59±2.46 |
| Tobacco intake, no. | 15 | 12 |
| Alcohol intake, no. | 30 | 26 |
| Antibiotics use, no. | 0 | 0 |
| Complications, no. |  |  |
| Diarrhea | 0 | 0 |
| Constipation | 0 | 0 |
| PANSS | 49.86±15.18 | - |

Note: BMI, body mass index; PANSS, positive and negative syndrome scale; SD, standard deviation.
